# Prediction of novel stable compounds in the Mg-Si-O system under exoplanet pressures

**DOI:** 10.1038/srep18347

**Published:** 2015-12-22

**Authors:** Haiyang Niu, Artem R. Oganov, Xing-Qiu Chen, Dianzhong Li

**Affiliations:** 1 Moscow Institute of Physics and Technology, 9 Institutskiy Lane, Dolgoprudny city, Moscow Region 141700, Russia; 2 Shenyang National Laboratory for Materials Science, Institute of Metal Research, Chinese Academy of Sciences, Shenyang 110016, China; 3 Skolkovo Institute of Science and Technology, Skolkovo Innovation Center, 3 Nobel St., Moscow 143026, Russia; 4 Department of Geosciences, Center for Materials by Design, and Institute for Advanced Computational Science, State University of New York, Stony Brook, NY 11794-2100.; 5 School of Materials Science, Northwestern Polytechnical University, Xi’an 710072, China

## Abstract

The Mg-Si-O system is the major Earth and rocky planet-forming system. Here, through quantum variable-composition evolutionary structure explorations, we have discovered several unexpected stable binary and ternary compounds in the Mg-Si-O system. Besides the well-known SiO_2_ phases, we have found two extraordinary silicon oxides, SiO_3_ and SiO, which become stable at pressures above 0.51 TPa and 1.89 TPa, respectively. In the Mg-O system, we have found one new compound, MgO_3_, which becomes stable at 0.89 TPa. We find that not only the (MgO)_x_·(SiO_2_)_y_ compounds, but also two (MgO_3_)_x_·(SiO_3_)_y_ compounds, MgSi_3_O_12_ and MgSiO_6_, have stability fields above 2.41 TPa and 2.95 TPa, respectively. The highly oxidized MgSi_3_O_12_ can form in deep mantles of mega-Earths with masses above 20 M_⊕_ (M_⊕_:Earth’s mass). Furthermore, the dissociation pathways of pPv-MgSiO_3_ are also clarified, and found to be different at low and high temperatures. The low-temperature pathway is MgSiO_3_ ⇒ Mg_2_SiO_4_ + MgSi_2_O_5_ ⇒ SiO_2_ + Mg_2_SiO_4_ ⇒ MgO + SiO_2_, while the high-temperature pathway is MgSiO_3_ ⇒ Mg_2_SiO_4_ + MgSi_2_O_5_ ⇒ MgO + MgSi_2_O_5_ ⇒ MgO + SiO_2_. Present results are relevant for models of the internal structure of giant exoplanets, and for understanding the high-pressure behavior of materials.

Several astonishing discoveries have been recently achieved in planetary science, e.g, the discovery of super-Earth Gliese 832c^1^.This planet weighs at least 5 M_⊕_(M_⊕_ : Earth’s mass) and is the nearest candidate for habitable planet so far; a new type of planet, Kepler-10c, weighing 17 times as much as Earth, is also found to be a rocky planet^2^. Such a planet was previously believed to be impossible to form, because anything so heavy would grab hydrogen gas as it grew, and become a Jupiter-like gas giant. For now, this planet is the biggest rocky planet ever discovered, much bigger than previously discovered “super-Earths” (with masses 1 to 10 M_⊕_), making it a “mega-Earth” (with masses over 10 M_⊕_)^2^. These breakthroughs emphasize the importance of the exploration of internal structure and mineralogy of super-Earths and mega-Earths.

After the mysterious anomalies in the Earth’s D” layer have been at least partly explained by the discovery of the new mineral phase post-perovskite (pPv) MgSiO_3_^3^,^4^, one wonders whether phase transitions exist in MgSiO_3_ under further compression, which is the key information to understand and model the internal structure of exoplanets. It was first reported that pPv-MgSiO_3_ will decompose into MgO and SiO_2_^5^ under high pressure. However, with prediction of two new high-pressure silicates, MgSi_2_O_5_^6^ and Mg_2_SiO_4_^7^, the dissociation pathway of pPv-MgSiO_3_ became a complex three-step process at zero Kelvin: pPv-MgSiO_3_ first decomposes into Mg_2_SiO_4_ and MgSi_2_O_5_ at 0.77 TPa, then MgSi_2_O_5_ breaks down into Mg_2_SiO_4_ and SiO_2_ at 1.25 TPa, eventually Mg_2_SiO_4_ dissociates into MgO and SiO_2_ at 3.09 TPa. However, the effect of temperature on stability of Mg_2_SiO_4_ and MgSi_2_O_5_, which is extremely important in exoplanet mantles, has not been considered.

Recently, numerous counterintuitive compounds have been discovered under pressure. For instance, in Li-H system, besides “normal” LiH, new “counterintuitive” compounds LiH_2_, LiH_6_ and LiH_8_ are predicted to be stable under pressure^8^; moreover, experimental synthesis and characterization confirm the existence of unexpected Na-Cl compounds (such as Na_3_Cl and NaCl_3_)^9^; what’s more, magnesium oxide (MgO), one of the most abundant phases in the Earth’s mantle, was long believed to be the only binary compound in the Mg-O system. Nevertheless, two extraordinary compounds, MgO_2_ and Mg_3_O_2_ have been discovered to be stable above 116 GPa and 500 GPa, respectively^10^. These fascinating discoveries inspired us to explore possible stable binary and ternary compounds in the Mg-Si-O system.

In this work, we have performed comprehensive structure searches and investigations of the Mg-Si-O system in the pressure range 0.5–3 TPa. Due to the complexities of the ternary system, the Mg-Si, Si-O and Mg-O bounding binaries are discussed first. All of the ternary stable compounds (including the stable compounds discovered in this work) fall into the pseudo-binary MgO-SiO_2_ and MgO_3_-SiO_3_ joins. Hence, we discuss ternary compounds in these two pseudo-binary systems separately. Lattice dynamics calculations for all the investigated structures show no imaginary vibrational frequencies, suggesting their dynamical stability throughout the pressure ranges reported here.

## Results and Discussions

Variable-composition structure searches using the USPEX code with up to 64 atoms in the unit cell at pressures ranging from 0.5 TPa to 3 TPa for the Mg-Si-O system have been carried out, identifying important low-energy structures that are likely to gain stability within this chemical system. Before we talk about binary and ternary compounds in the Mg-Si-O system, crystal structures of elemental Mg, Si and O should be clarified. For elemental Mg, several phase transitions are predicted in the pressure range 0.5–3 TPa. In excellent agreement with previous studies^10^,^11^, our calculations demonstrate that Mg adopts the fcc structure at 0.5 TPa, then it transforms into the simple hexagonal (sh) structure at 0.76 TPa; interestingly, when pressure increases to 1.07 TPa, it transforms into the simple cubic (sc, or α-Po) structure. Elemental Si adopts the fcc structure at 0.5 TPa, in agreement with literature^12^, but no phase transformation occurs in the pressure range of 0.5–3 TPa. Elemental O adopts a hexagonal *hP*8 structure at 0.5 TPa (Several similar structures are very close in enthalpy in the pressure range of 0.5 to 1.9 TPa. For more details, we refer to Ref. 13) and then transforms into the orthorhombic *oC*16 structure at 1.9 TPa, in good agreement with literature^13^.

### Mg-Si binary system

Mg_2_Si is the only binary compound in the Mg-Si system at ambient pressure^14^. When pressure is increased above 0.5 TPa, Mg_2_Si remains the only stable binary compound in the Mg-Si system, until it decomposes into Mg and Si at 1.41 TPa (see Fig. S3a in Supplementary Materials). In this pressure range, it adopts the well-known AlB_2_-type structure (Fig. S1b).

### Si-O binary system

**Figure 1:**
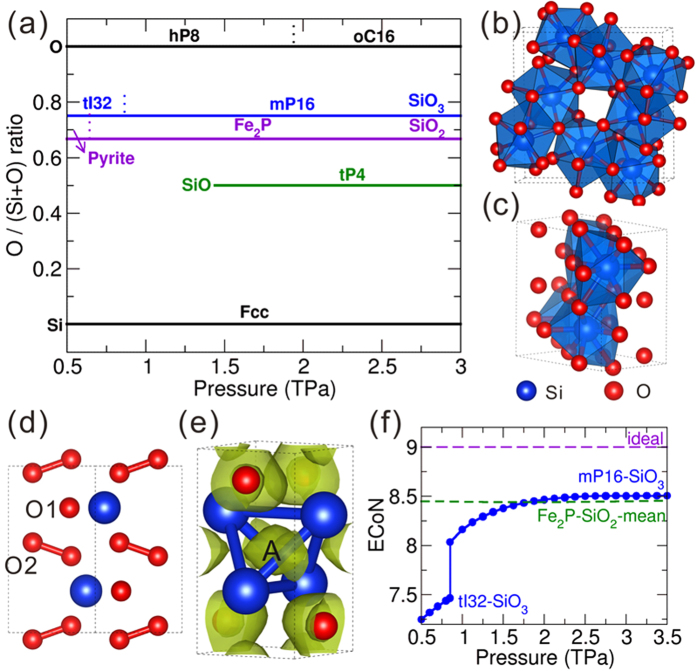
(**a**) Pressure-composition phase diagram of the Si-O system. Crystal structures of (**b**) *tI*32-SiO_3_ and (**c**,**d**) *mP*16-SiO_3_. O1 and O2 refer to two types of O atoms in *mP*16-SiO_3_. (**e**) Crystal structure of *tP*4-SiO and isosurface of the electron localization function (ELF) with an isovalue of 0.65. Letter A refers to the strong interstitial electronic attractor in the Si_4_ tetrahedron. (**f**) ECoN for *tI*32-SiO_3_ and *mP*16-SiO_3_ as a function of pressure. The mean ECoN value for Fe_2_P-SiO_2_ is shown by a green dashed line, and the ideal CoN of 9 is given by a purple dashed line. The densities of states of *tI*32-SiO_3_, *mP*16-SiO_3_, and *tP*4-SiO show that they are insulators at 0 K, see Supplementary Materials.

Even though silicon monoxide SiO can exist in the gas phase^15^, no evidence shows that it can exist in the crystalline form, and the amorphous black solid form of silicon monoxide indeed is a mixture of amorphous silicon and silicon dioxide^15^. Therefore, silicon dioxide SiO_2_ is still the only known oxide in the Si-O system. In agreement with previous work^16^, pyrite-type SiO_2_ transforms into the Fe_2_P-type phase at 0.69 TPa. Nevertheless, if crystal structure exploration is carried out in the entire Si-O binary system, some unforeseeable structures are found. Figure 1a demonstrates the pressure-composition phase diagram of the Si-O system. A new oxide, SiO_3_, becomes thermodynamically stable at 0.51 TPa with the *tI*32 (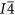) structure. Interestingly, this *tI*32-SiO_3_ can further transform into the *mP*16 (*P*2_1_/*c*) structure at 0.82 TPa. As illustrated in Fig. 1b,c, both structures can be constructed by SiO_9_ polyhedra (tricapped trigonal prisms), which is exactly the same coordination polyhedron as in Fe_2_P-type SiO_2_^16^.

In order to further distinguish polyhedra in the two structures of SiO_3_, effective coordination numbers (ECoN)^17^ have been calculated. A large increase of the ECoN at the phase transition point from *tI*32 (ECoN = 7.48) to *mP*16 (ECoN = 8.05) phase can be observed in Fig. 1f, indicating that accommodation of increased coordination is the primary reason for the stability of *mP*16-SiO_3_ compared to *tI*32-SiO_3_. When pressure increases further, the ECoN of *mP*16-SiO_3_ reaches 8.5, equal to the mean value of the SiO_9_ polyhedron in Fe_2_P-SiO_2_^16^. Perhaps surprisingly, the Si-O distances are in the range from 1.53 to 1.95 Å in *tI*32-SiO_2_ and 1.54 to 1.82 Å in *mP*16-SiO_3_ at 0.7 TPa, respectively. These distances are unexpectedly long under such a high pressure, and comparable to the values (1.6 Å) in silica and silicates at ambient pressure. This phenomenon is partly a consequence of geometry, since the typical bond-length must increase in order to accommodate the dramatic increase in Si-O coordination. Therefore, the relative Si-O bond length must necessarily increase with increasing coordination as the bonding polyhedra’s size expands to fill the space, a general phenomenon that is well-represented by a recently proposed coordinated hard sphere mixture model^18^. The same situation is also observed in Fe_2_P-SiO_2_^16^, which indicates the tendency to form highly coordinated structures instead of shrinking the Si-O distances to lower the system energy.

When pressure is raised further, stable solid silicon monoxide appears in the Si-O system with the *tP*4 structure (*P*4/*nmm*) at 1.89 TPa, see Fig. 1d and Fig. S5c. SiO crystallizes in a layered structure with Si-Si-O-O stacking order. Each Si atom is coordinated by five O atoms and eight Si atoms. Therefore, SiO retains high coordination numbers, like SiO_2_ and SiO_3_, despite the drop of oxygen content.

SiO_3_ and SiO are both dynamically and thermodynamically stable, and it is still puzzling what stabilizes these exotic compounds. Based on classical chemical valence, only SiO_2_ can be expected. To unravel the nature of these new phases, their electronic structure and chemical bonding have been analyzed.

As *tI*32-SiO_3_ and *mP*16-SiO_3_ display similar charge transfer and chemical bonding features, *mP*16-SiO_3_ has been selected for the following discussion. In *mP*16-SiO_3_ at 1 TPa, the net Bader charge^19^,^20^ on Si is +3.42 *e*, indicating a very large degree (~85%) of charge transfer from Si to O atoms. Based on Bader analysis, two types of O atoms exist in the *mP*16-SiO_3_ structure (Fig.1d), the net charges on O1 and O2 are −1.63 *e* and −0.89 *e*, respectively. Therefore O1 attracts almost two electrons and attains a stable s^2^p^6^ electron configuration. Furthermore, the O-O bond distance between O2 atoms is 1.19 Å, the O-O bond distance for molecular crystal *hP*8-O_2_ at 1 TPa is 1.09 Å while the non-bonding O-O distances for MgSiO_3_ and SiO_2_ are in the range of 1.7 Å to 2.0 Å, which clearly indicates a covalent bond and the presence of a peroxide-ion [O-O]^2−^, fulfilling the octet rule. Electron Localization Function (ELF)^21^ of *mP*16-SiO_3_(Fig. S5b) confirms these conclusions: O2 atoms form peroxo-groups, while O1 atoms do not. SiO_3_ can be classified as a “peroxide oxide”, with a structural formula SiO[O_2_], just like the recently predicted Al_4_O_7_ and AlO_2_^22^, in which O^2−^ and [O_2_]^2−^ ions are simultaneously present.

For *tP*4-SiO at 1.5 TPa, the net charge on Si is +1.83 *e*, and the net charge on O is −1.83 *e*. Thus, O atom attains a stable closed-shell electronic configuration. ELF distribution of *tP*4-SiO shows that besides accumulated electrons surrounding O atoms, we can also observe a strong interstitial electron localization in the Si_4_ tetrahedron as marked by letter A in Fig.1e. Considering the Si-Si distance (1.86 Å) is out of the range of core-core orbital overlap, the strong interstitial electron localization is due to the formation of multicenter covalent bonds between Si atoms. Each Si atom has four nearest such electron localization regions, each of which accumulates two valence electrons, indeed creating an octet and explaining why each Si atom can be stabilized with two valence electrons and why SiO adopts a Si-Si-O-O ordered layered structure.

### Mg-O binary system

**Figure 2:**
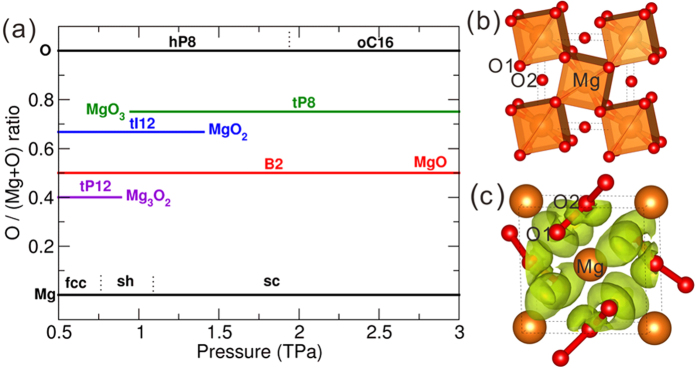
(**a**) Pressure-composition phase diagram of the Mg-O system and illustration of (**b**) crystal structure of *tP*8-MgO_3_ and (**c**) its isosurface of the electron localization function (ELF) with an isovalue of 0.65. O1 and O2 refer to two types of O atoms in *tP*8-MgO_3_. All Mg oxides are insulators at 0 K, see Supplementary Materials.

Besides MgO, two novel stochiometries MgO_2_ and Mg_3_O_2_ have recently been found to be stable under high pressure in the Mg-O system^10^. Intriguingly, if we further increase pressure, another extraordinary compound, *tP*8-MgO_3_ with 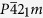 symmetry, becomes thermodynamically stable at 0.89 TPa as shown in the pressure-composition phase diagram of the Mg-O system (Fig. 2a). Furthermore, Mg_3_O_2_ decomposes into MgO and Mg at 0.95 TPa, while MgO_2_ decomposes into MgO and MgO_3_ at 1.43 TPa, and above 1.43 TPa MgO_3_ and MgO are the only two stable magnesium oxides.

As shown in Fig. 2b, each Mg atom within MgO_3_ has 8 nearest O neighbors (O1 atoms) forming a cubic coordination (just as in B2-MgO) and 4 second nearest O neighbors (O2 atoms). Mg and O1 atoms form a distorted fluorite-type structure, empty voids of which are stuffed with O2 atoms. According to Bader analysis, in *tP*8-MgO_3_ at 1 TPa the net charge on Mg is +1.75 *e*, indicating the nearly complete transfer of valence electrons of Mg to O atoms. The net charges on O1 and O2 are −0.74 *e* and −0.18 *e*, respectively, while the Mg-O1 and Mg-O2 distances are 1.63 Å and 1.83 Å, respectively. Considering the O-O distance between O1 and O2 is 1.22 Å,and the O-O bond distance for molecular crystal *hP*8-O_2_ at 1 TPa is 1.09 Å while the non-bonding O-O distances for MgSiO_3_ and SiO_2_ are in the range of 1.7 Å to 2.0 Å, we can conclude that two O1 atoms and one O2 atoms form a bent singly bonded [O-O-O]^2−^ group. From the ELF isosurface of *tP*8-MgO_3_ illustrated in Fig. 2c, we can also confirm the existence of [O-O-O]^2−^, with a significant electronic accumulation between O1 and O2 atoms. As far as we know, this type of trioxide group is found here for the first time.

### Mg-Si-O ternary system

**Figure 3:**
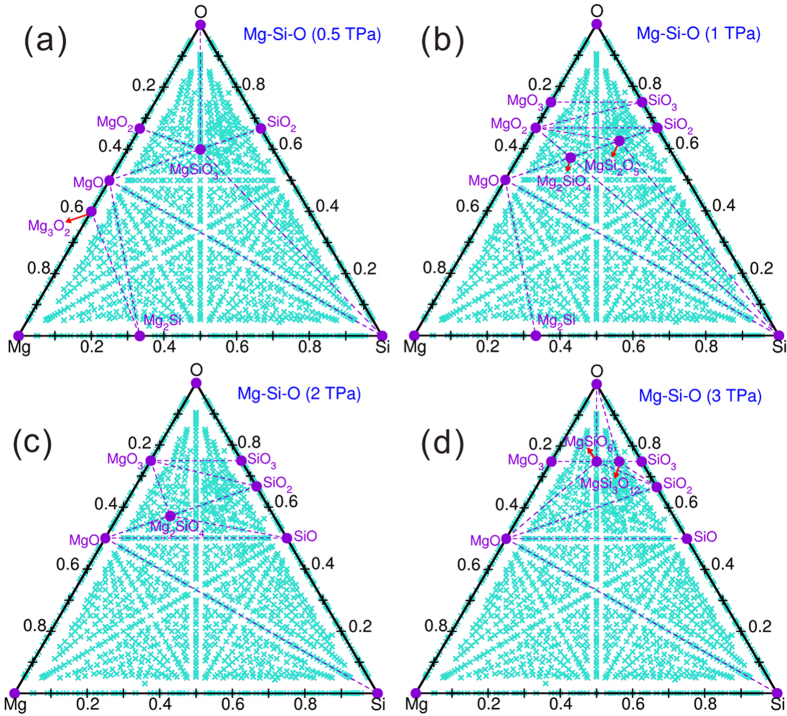
Mg-Si-O phase diagram at (a) 0.5 TPa, (b) 1 TPa, (c) 2 TPa and (d) 3 TPa, respectively.

Phase diagrams of the Mg-Si-O ternary system in the pressure range 0.5–3 TPa, obtained through variable-composition crystal structure prediction for the ternary system, are shown in Fig. 3. In excellent agreement with previous works^6^,^7^, Mg_2_SiO_4_ and MgSi_2_O_5_ become thermodynamically stable under high pressure. We have also found two new stable ternary compounds, MgSiO_6_ and MgSi_3_O_12_. The MgO-SiO_2_and MgO_3_-SiO_3_pseudo-binaries contain numerous important stable compounds and are discussed in detail below.

### MgO-SiO_2_ pseudo-binary system

**Figure 4:**
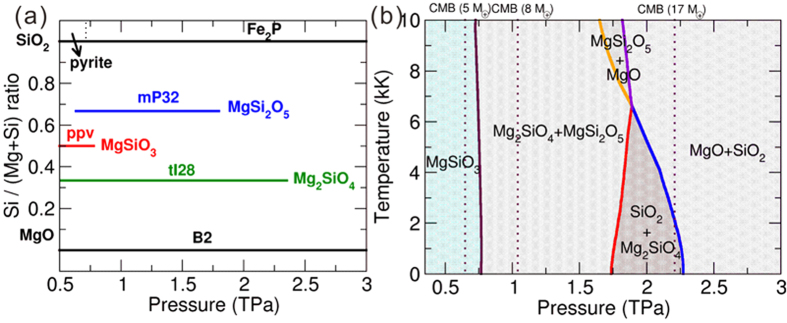
(**a**) Pressure-composition phase diagram of the pseudo-binary MgO-SiO_2_ system. (b) *P-T* phase diagram of MgSiO_3_. The core-mantle boundary (CMB) pressures of super-Earths and mega-Earths with 5, 8 and 17 M_⊕_ are also plotted by vertical dashed lines.

In good agreement with previous works^6^,^7^, Mg_2_SiO_4_ with the *tI*28 (*I*
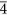
2*d*) structure and MgSi_2_O_5_ with the *mP*32 (*P*2_1_/*c*) structure become thermodynamically stable at 0.51TPa and 0.63 TPa, respectively, as shown in Fig. 4a. With increasing pressure, at zero Kelvin pPv-MgSiO_3_ decomposes into Mg_2_SiO_4_ and MgSi_2_O_5_ at 0.79 TPa, and then MgSi_2_O_5_ decomposes into Mg_2_SiO_4_ and SiO_2_ at 1.80 TPa. Mg_2_SiO_4_, the last ternary compound in the MgO-SiO_2_ pseudo-binary system, eventually decomposes into MgO and SiO_2_ at 2.3 TPa.

Temperature, another important factor affecting stability of minerals, should be considered when developing models of the internal structure of exoplanets. Here, thermodynamic properties of these phases were investigated within the quasiharmonic approximation (QHA), using the computed phonon spectra. Previous work suggests that the *P*-*T* conditions of interest are within the range of validity of the QHA^5^,^23^. The *P*-*T* phase diagram of MgSiO_3_, as shown in Fig. 4b, is determined by comparing finite-temperature Gibbs free energies of relevant phases and phase assemblages.

In order to evaluate the electronic entropy contribution, we have calculated the electronic structures and phonon dispersions of these newly reported compounds at finite temperatures (2 kK, 5 kK, 10 kK) within the Fermi-Dirac-smearing approach^24^. We have found that all the compounds discussed in Fig. 4b show very small electronic effects at these temperatures. For instance, for the decomposition reaction of MgSiO_3_ into Mg_2_SiO_4_ and MgSi_2_O_5_ under 0.75 TPa at 10 kK, the enthalpy changes by only 0.0006 eV/atom after taking electronic entropy into consideration, and the *dP*/*dT* slope of this reaction in Fig. 4b becomes more negative, but the change is so tiny that we can safely neglect the electronic entropy contribution. Other reactions in Fig. 4b show similar behavior. In order to further understand this question, we have calculated the band gaps of these compounds under different pressures as listed in Table S1 in the supplementary materials. We can observe that all the compounds discussed in Fig. 3d (MgO, SiO_2_, MgSiO_3_, Mg_2_SiO_4_, MgSi_2_O_5_) show wide band gaps, and the electronic structures and their phonon frequencies are not affected significantly by high temperature.

As shown in Fig. 4b, the dissociation pathways of pPv-MgSiO_3_ are different at high and low temperatures. At high temperatures (>6,610 K), MgSiO_3_ decomposes into Mg_2_SiO_4_ and MgSi_2_O_5_, followed by decomposition of Mg_2_SiO_4_ into MgO and MgSi_2_O_5_. The last stable ternary compound in the MgO-SiO_2_ pseudo-binary system is MgSi_2_O_5_, it eventually decomposes into MgO and SiO_2_ at relatively high temperature well within the *P-T* range of mega-Earth mantles. This decomposition pathway is most likely for giant exoplanets and has not been reported before. These phase transitions and reactions are expected to impact the dynamics of exoplanet interiors: as exothermic transformations (*dP*/*dT* > 0) generally enhance heat transfer through convection, while endothermic transformations (*dP*/*dT* < 0) decrease it^25^.As shown in Fig. 4b, decomposition of MgSi_2_O_5_ to Mg_2_SiO_4_ and SiO_2_ holds positive *dP*/*dT* slope, and should thus enhance convection, while all other transformations shown in Fig. 4b hold negative *dP*/*dT* slopes, partially inhibiting convection.

### MgO_3_-SiO_3_ pseudo-binary system

MgSiO_3_, Mg_2_SiO_4_ and MgSi_2_O_5_ are traditional ordinary compounds satisfying the composition (MgO)_x_·(SiO_2_)_y_ (x, y: positive integers).The discovery of novel compounds MgO_3_,SiO_3_ and SiO suggests that other compositions may appear in the ternary system. Excitingly, we have discovered two new stable magnesium silicates which belong to the MgO_3_-SiO_3_ pseudo-binary system.

**Figure 5:**
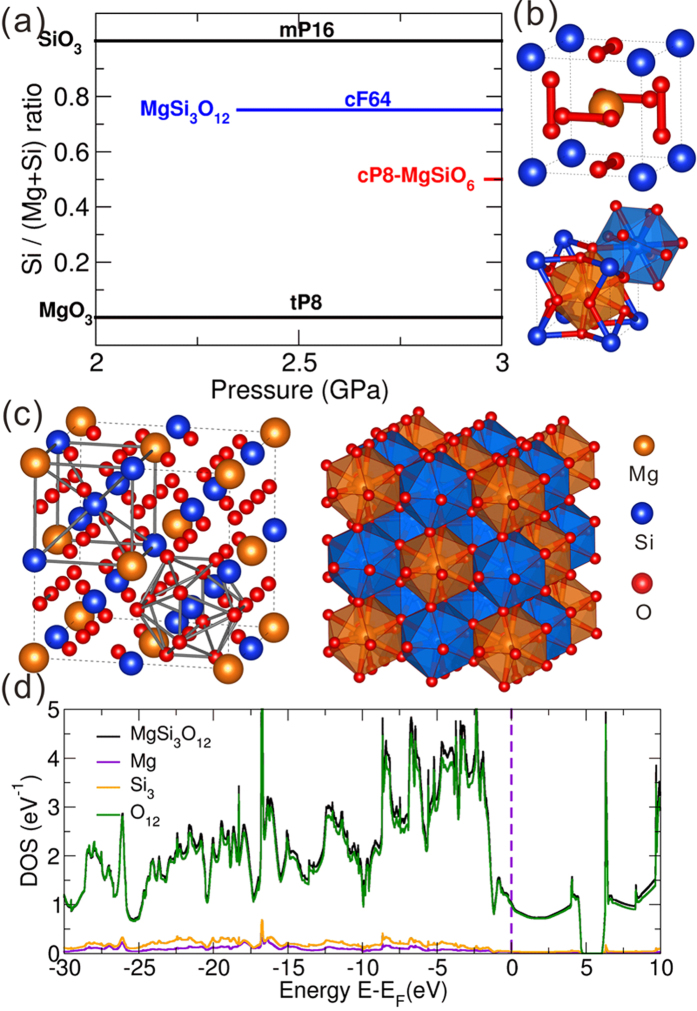
(**a**) Pressure-composition phase diagram of the pseudo-binary MgO_3_-SiO_3_ system and crystal structures of (**b**) MgSiO_6_ and (**c**) MgSi_3_O_12,_ and (**d**) density of states (DOS) of *cF*64-MgSi_3_O_12_. The density of states of *cP*8-MgSiO_6_ is shown in Fig. S8 in Supplementary Materials.

As shown in Fig. 5a, MgSi_3_O_12_ with 64 atoms in the unit cell and *cF*64(*Fm*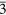) structure becomes stable at 2.41 TPa. By increasing pressure further, another ternary compound, MgSiO_6_ (*cP*8, *Pm*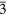) gains stability at 2.95 TPa. The two compounds share many similar structural features, as illustrated in Fig. 5b,c. Both are ordered cubic superstructures of the Cr_3_Si-type structure. Recently^9^ we have discovered a novel compound NaCl_3_ with the Cr_3_Si-type structure, and a related compound NaCl_7_. This structure is stable under pressure because of high density and high coordination numbers. Mg and Si atoms in MgSiO_6_ and MgSi_3_O_12_ are both icosahedrally coordinated (CN and ECoN = 12).

For these new ternary magnesium silicates, we need to clarify the nature of their stability. In both compounds, one can see infinite non-intersecting O-chains along the x, y and z axes. The O-O distances in *cF*64-MgSi_3_O_12_ are in the range 1.29–1.33 Å, which are much longer than in MgSiO_6_. Taking into account the O-O bond distance of *oI*16-O at 3 TPa is 1.10 Å, we can conclude that the O-O bonding in *cF*64-MgSi_3_O_12_ are much weaker than covalent single O-O bond. From Bader analysis, for *cP*8-MgSiO_6_ at 3 TPa, the net charge on Mg and Si are +1.59 *e* and +3.48 *e*, respectively, while the net charge on O is −0.85 *e*, indicating the nearly complete transfer of valence electrons of Mg and Si atoms to O atoms. For *cF*64-MgSi_3_O_12_ at 3 TPa, the net charges on Mg and Si are +1.6 *e* and +3.49 *e*, respectively, i.e. practically the same values as in *cP*8-MgSiO_6_, while the charge on O is −1.01 *e*, which is much higher than the value (−0.85*e*) of O atom in *cP*8-MgSiO_6_. The density of states of *cF*64-MgSi_3_O_12_ (Fig. 5d) shows that MgSi_3_O_12_ is a metal, with DOS near the Fermi level exhibiting features of a 1D-metal, which is consistent with the infinite non-intersecting O-chains in this structure. It is worth emphasizing that all the other oxides discussed in this work are insulators, which demonstrates the unique electronic structure of *cF*64-MgSi_3_O_12_.

**Figure 6:**
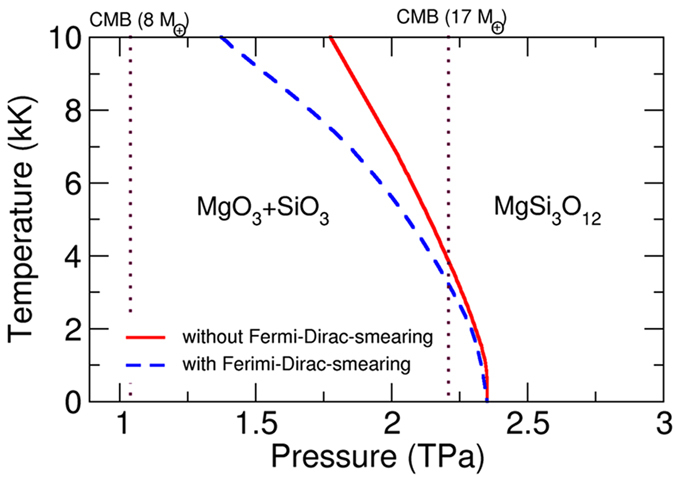
*P-T* phase diagram of MgSi_3_O_12_. The red and dotted blue lines refer to the phase boundary lines with and without Fermi-Dirac-smearing, respectively. The core-mantle boundary (CMB) pressures of super-Earths and mega-Earths with 8 and 17 M_⊕_ are also plotted by vertical dashed lines.

By adopting Fermi-Dirac-smearing approach^24^, we have found that the electronic entropies of MgSiO_6_ and MgSi_3_O_12_ are much more significant and can’t be neglected. For instance, the enthalpy changes 0.10 eV/atom for MgSiO_6_ under 3.0 TPa at 10 kK after taking electronic entropy into account. MgSiO_6_ behaves more like a semi-conductor with band gap of 1.49 eV under 3.0 TPa, therefore bottom of the conduction band of MgSiO_6_ becomes populated and the phonon frequencies changes at high temperature. This effect is even larger for MgSi_3_O_12_ since MgSi_3_O_12_ is a metal, the enthalpy changes 0.11 eV/atom for MgSi_3_O_12_ under 2.0 TPa at 10 kK after taking electronic entropy into account. Here we have calculated the P-T phase diagram of MgSi_3_O_12_ with and without the Fermi-Dirac-smearing. As shown in Fig. 6, the reaction from MgO_3_ and SiO_3_ to MgSi_3_O_12_ is affected significantly by electronic entropy, and the phase boundary line shifts toward lower pressures. For Fig. 6, we can also observe that the stability of MgSi_3_O_12_ increases with increasing temperature. For O-rich exoplanets, MgSi_3_O_12_ are expected to exist at high temperature and pressure. It’s worth emphasizing that MgSiO_6_ is not stable below 3.0 TPa after considering zero-point energy, that’s why MgSiO_6_ cannot be observed in Fig. 6. Furthermore, for metallic and semiconducting compounds predicted in this work (MgSiO_6_, MgSi_3_O_12_), there is an intriguing possibility of their enhanced solubility in metallic iron-rich cores of exoplanets.

## Conclusions

Using first-principles calculations and variable-composition evolutionary structure exploration in the Mg-Si-O system under exoplanet pressures, we have discovered numerous unexpected compounds. Two extraordinary compounds, SiO_3_ and SiO, have been found to become stable at pressures above 0.51 TPa and 1.89 TPa, respectively, in the Si-O system. Both *tI*32 and *mP*16 forms of SiO_3_ are peroxide oxides containing oxide O^2−^ and peroxide [O_2_]^2−^ ions, while strong electron localization in the Si_4_-tetrahedron plays the role of an additional anion to stabilize *tP*4-SiO. Besides two previously reported unusual compounds MgO_2_ and Mg_3_O_2_, we have found another extraordinary compound, *hP*8-MgO_3_, in the Mg-O system, which becomes thermodynamically stable at 0.89 TPa.

Taking temperature into consideration, two dissociation pathways of MgSiO_3_ are found at relatively low (<6.4 kK) and high (>6,6 kK) temperature are:


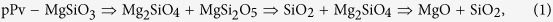



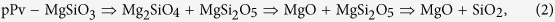


respectively. Interestingly, besides the well-known (MgO)_x_·(SiO_2_)_y_ compounds, we have discovered two (MgO_3_)_x_·(SiO_3_)_y_ compounds, MgSi_3_O_12_, MgSiO_6_, which can form at 2.41 TPa and 2.95 TPa, respectively, in the Mg-Si-O system. Surprisingly, MgSi_3_O_12_ is predicted to be a metallic oxide with 1D-metalicity while all other oxides discussed in this work are semiconductors or insulators.

As the dissociation pathway of pPv-MgSiO_3_ is clarified, the mineralogy and internal structure of planetary mantles can be understood much deeper. pPv-MgSiO_3_ can survive in super-Earths with masses smaller than 6 M_⊕_ as shown in Fig. 6b. Mg_2_SiO_4_ and MgSi_2_O_5_ can be found in the mantle of super-Earths with masses larger than 6 M_⊕_. Kepler-10c, 17 times heavier than Earth, would probably only have binary MgO and SiO_2_ near the CMB. For strongly oxidized planets, MgO_3_ and SiO_3_ can be expected to be found. The newly discovered MgO_3_, SiO_3_, MgSiO_6_, MgSi_3_O_12_ hold non-traditional stoichimetries, which fall off the MgO-SiO_2_ binary system. Given their thermodynamic stability, these new compounds must be included in future models of exoplanet mineralogy in order to better understand the role that they play in massive planetary structure and evolution. The highly-oxidized MgSi_3_O_12_ can be formed in the lowermost mantles of mega-Earths with masses above 20 M_⊕_, and even a metallic layer can exist. For O-rich planets, the extraordinary O-rich compounds MgO_3_, SiO_3_, MgSi_3_O_12_ and perhaps MgSiO_6_ can be important planet-forming minerals. They may also appear in gas giants, as a result of reaction between Mg-silicate solid core and H_2_O-rich fluid mantle. In future, the consideration of other important elements (e.g., Fe, Al), will likely reveal additional important high-pressure phases with similarly strange stoichiometries.

Further models of the internal structures of exoplanets must take these findings into account. Phase transitions and reactions predicted here will have a profound effect not only on the internal structure, but also on dynamical processes in planets. Exothermic reactions (with positive Clapeyron slope *dP/dT* in Fig. 4b) enhance convection, endothermic ones slow down or stop it, and a metallic layer can affect the planetary magnetic field^25^,^26^. Structure, dynamics and chemistry of planetary interiors may be much more complex and surprising than previously thought.

## Computational Methods

Searches for stable compounds and structures were performed using the variable-composition evolutionary algorithm, as implemented in the USPEX code^27^,^28^,^29^,^30^,^31^ merged with first-principles calculations within the framework of density functional theory (the Vienna Ab initio Simulation Package VASP)^32^,^33^ for the calculation of the total energies, structure relaxation, and computing their electronic structures. The electronic structure and force calculations at finite temperatures were implemented within the Fermi-Dirac-smearing approach^24^. The most significant feature of USPEX we used in this work is the capability of optimizing the composition and crystal structures simultaneously - as opposed to the more usual structure predictions at fixed chemical composition. The compositional search space is described via chemical building blocks. The whole range of compositions of interest is initially sampled randomly and sparsely. To ensure the child structures are within the desired area of compositional space, the chemistry-preserving constraints in the variation operators are lifted and replaced by the block correction scheme. A special “chemical transmutation” is introduced to reinforce the search efficiency. Stable compositions are determined using the convex hull construction: a compound is thermodynamically stable if the enthalpy of its decomposition into any other compounds is positive. For first-principles calculations we employed the all-electron projector augmented wave(PAW) method^34^ and the generalized gradient approximation^35^ for the exchange-correlation energy, along with a plane-wave cutoff energy of 800 eV and dense uniform Γ-centred k-point meshes with a reciprocal space resolution of 2π × 0.03 Å^−1^. The PAW potentials have [He] cores for all atoms, with radii 1.25, 1.4 and 1.15 a.u. for Mg, Si and O, respectively, which can guarantee no core overlap even at the highest pressures studied here. In addition, phonon dispersions throughout the Brillouin zone were derived using the finite-displacement approach as implemented in the Phonopy code^36^. Thermodynamic properties of these phases were investigated using their phonon spectra within the quasiharmonic approximation (QHA).

## Additional Information

**How to cite this article**: Niu, H. *et al.* Prediction of novel stable compounds in the Mg-Si-O system under exoplanet pressures. *Sci. Rep.* **5**, 18347; doi: 10.1038/srep18347 (2015).

## Supplementary Material

Supplementary Information
