# Supplementary material for: Prediction of novel stable compounds in the Mg-Si-O system under exoplanet pressures
Source: Sci Rep. 2015 Dec 22;5:18347. doi: 10.1038/srep18347 (PMC4686916; doi:10.1038/srep18347)
Supplement: Supplementary Information [file srep18347-s1.pdf]

SUPPLEMENTARY MATERIALS

Prediction of novel stable compounds in the Mg-Si-O system under exoplanet pressures

Haiyang Niu[1,2], Artem R. Oganov[3, 1, 4, 5, *], Xing-Qiu Chen[2,**], and Dianzhong Li[2]

[1]*Moscow Institute of Physics and Technology, 9 Institutskiy Lane,*

Dolgoprudny city, Moscow Region 141700, Russia

[2]*Shenyang National Laboratory for Materials Science, Institute of Metal Research,*

Chinese Academy of Sciences, Shenyang 110016, China

[3] *Skolkovo Institute of Science and Technology, Skolkovo Innovation Center, 3 Nobel St., Moscow 143026, Russia*

[4] *Department of Geosciences, Center for Materials by Design, and Institute for Advanced Computational Science, State University of New York, Stony Brook, NY 11794-2100.*

[5] *School of Materials Science, Northwestern Polytechnical University, Xi'an 710072, China*

(Dated: October 11, 2015)

* *artem.oganov@stonybrook.edu* ** *xingqiu.chen@imr.ac.cn*

SUPPLEMENTARY TABLE and FIGURES

1. Table S1 Calculated space groups, lattice parameters, Wyckoff positions and band gaps of the investigated compounds in the Mg-Si-O system.
2. Fig. S1. (a) Pressure-composition phase diagram of the Mg-Si system. (b) and (c) Crystal structure of Mg_2Si.
3. Fig. S2. The density of states (DOS) of Mg_2Si at 0.5 TPa.
4. Fig. S3. (a) Crystal structure and (b) its corresponding isosurface of the electron localization function (ELF)of *mP*16-SiO_3. (c) Crystal structure of *tP*4-SiO with Si-Si-O-O layered structure.
5. Fig. S4. The density of states (DOS) of *tP*4-SiO at 1.5 TPa, *tI*32-SiO_3 at 0.7 TPa, and *mP*16-SiO_3 at 1.0 TPa, respectively.
6. Fig. S5. The density of states (DOS) of *tP8*-MgO_3 at 1.0 TPa.
7. Fig. S6. The density of states (DOS) of Mg_2Si at 0.5 TPa.
8. Fig. S7. Cross-section of the ELF of (a) *cP*8-$MgSiO_6$ and (b) *cF*64-$MgSi_3O_{12}$

Table S1. Calculated space groups, lattice parameters, Wyckoff positions and band gaps of the investigated compounds in the Mg-Si-O system.

Phase	Pearson symbol	Space group	Cell parameters (Å)	Atom	Wyckoff position	x	y	z	Pressure （TPa)	Band gap (eV)
Mg-fcc	*cF*4	$Fm\bar{3}m$	*a*=2.952	Mg	4*a*	0.0	0.0	0.0	0.5	-
Mg-sh	*hP*1	*P6/mmm*	*a*=1.827<br>*c*=1.599	Mg	1*a*	0.0	0.0	0.0	1.0	-
Mg-sc	*cP*1	$Pm\bar{3}m$	*a*=1.536	Mg	1*a*	0.0	0.0	0.0	1.5	-
Si-fcc	*cF4*	$Fm\bar{3}m$	*a*=2.679	Si	4*a*	0.0	0.0	0.0	1.0	-
O	*hP*8	$P6_3/mmc$	*a*=1.704	O	4*e*	0.0	0.0	0.0537	1.0	-
			c=10.06	O	4*f*	0.3333	0.6667	0.3037		
O	*oI*16	$I4_1/acd$	*a*=4.46<br>*c*=1.707	O	16*f*	0.1344	0.1344	0.25	3.0	-
Mg_2Si	*hP*3	*P*6/*mmm*	*a*=3.021	Mg	1*a*	0.0	0.0	0.0	1.0	-
			c=1.852	Si	2*d*	0.3333	0.6667	0.5		
SiO	*tP*4	*P*4/*nmm*	*a*=1.926	Si	2*c*	0.0	0.5	0.6816	1.5	2.22
			c=3.500	O	2*c*	0.0	0.5	0.1414	3.0	1.93
SiO_2-Pyrite	*cP*12	$Pa\bar{3}$	*a*=3.731	Si	4*b*	0.5	0.5	0.5	0.5	11.09
				O	8*c*	0.1483	0.3517	0.6483		
SiO_2-Fe_2P	*hp*9	$P\bar{6}2m$		Si1	1*a*	0.0	0.0	0.0	1.0	5.43
			a=4.058	Si2	2*d*	0.3333	0.6667	0.5	2.0	4.09
			c=2.170	O1	3*g*	0.7429	0.0	0.5	3.0	2.75
				O2	3*f*	0.4093	0.0	0.0		
SiO_3	*tI*32	$I\bar{4}$		Si	8*g*	0.0613	0.2830	0.9960		
			a=6.554	O1	8*g*	0.0742	0.3769	0.49	0.7	3.73
			c=2.795	O2	8*g*	0.7875	0.85766	0.3713	0.8	3.57
				O3	8*g*	0.87	0.918	0.739		

SiO_3	*mP*16	$P2_1/c$	*a*=2.758	Si	4*e*	0.1029	0.2618	0.4003	1.0	4.34
			b=4.810	O1	4*e*	0.6999	0.2631	0.55	2.0	3.47
			c=4.421	O2	4*e*	0.7257	0.9592	0.7931	3.0	2.63
			β=113.66°	O3	4*e*	0.7200	0.9570	0.3189		
Mg_3O_2	*tP*12	*P4/mcm*	*a*=4.508	Mg1	2*a*	0.0	0.0	0.0	0.5	3.20
			c=2.367	Mg2	4*g*	0.3494	0.1506	0	0.75	3.19
				O	4*g*	0.8468	0.6532	0.0		
MgO	*cP*2	$Pm\bar{3}m$	*a*=2.052	Mg	1*a*	0.0	0.0	0.0	0.5	9.08
									1.0	8.97
				O	1*b*	0.5	0.5	0.5	2.0	8.94
									3.0	8.61
MgO_2	*tI*12	*I4/mcm*	*a*=3.377	Mg	4*a*	0.0	0.0	0.25	0.5	7.45
			c=3.985	O	8*h*	0.1260	0.3740	0.0	1.0	6.49
MgO_3	*tP*8	$P\bar{4}2_1m$	*a*=3.619	Mg	2*a*	0.0	0.0	0.0	1.0	5.68
			c=2.025	O1	4*e*	0.6993	0.1993	0.9801	2.0	6.41
				O2	2*c*	0.5	0.0	0.6475	3.0	6.67
$MgSiO_3$-pPv	*oC*20	*Cmcm*	*a*=2.225	Mg	4*c*	0.0	0.7487	0.25	0.5	8.75
			b=7.012	Si	4*a*	0.0	0.0	0.0	1.0	6.21
			c=5.566	O1	4*c*	0.0	0.0706	0.25	2.0	4.12
				O2	8*f*	0.0	0.6434	0.0605		
Mg_2SiO_4	*tI*28	$I\bar{4}2d$	*a*=4.686	Mg	8*d*	0.3872	0.25	0.125	1.0	10.69
			c=4.8356	Si	4*a*	0.0	0.0	0.0	2.0	10.55
				O	16*e*	0.9312	0.6868	0.9523	3.0	10.09
$MgSi_2O_5$	*mP*32	$P2_1/c$		Mg	4*e*	0.5055	0.5641	0.3368		
			a=7.662	Si	4*e*	0.2574	0.9309	0.2658	1.0	8.10
			b=4.051	Si	4*e*	0.9968	0.9507	0.3209	2.0	7.91
			c=6.208	O1	4*e*	0.2397	0.8254	0.0199	3.0	7.70
			β=144.47°	O2	4*e*	0.2338	0.1545	0.4285		
				O3	4*e*	0.2279	0.9675	0.7277		

				O4	4*e*	0.5424	0.8103	0.6207		
				O5	4*e*	0.0391	0.3270	0.4533		
$MgSiO_6$	*cP*8	$Pm\bar{3}$	*a*=2.621	Mg	1*b*	0.5	0.5	0.5	3.0	1.49
				Si	1*a*	0.0	0.0	0.0	3.5	1.06
				O	6*g*	0.2794	0.5	0.0		
$MgSi_3O_{12}$	*cF*64	$Fm\bar{3}$	*a*=5.247	Mg	4*a*	0.0	0.0	0.0		
				Si1	8*c*	0.25	0.25	0.25	2.0	-
				Si2	4*b*	0.5	0.5	0.5	3.0	-
				O	48*h*	0.0	0.1231	0.2512		

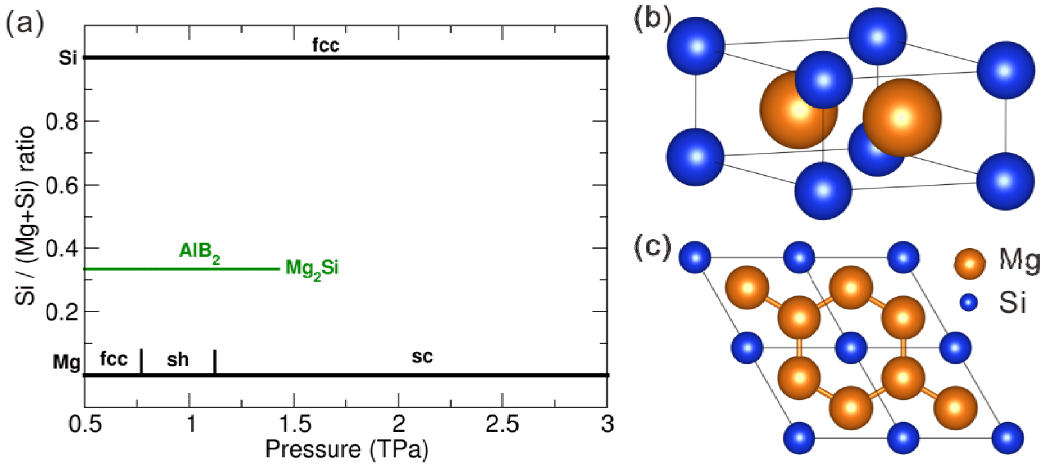

Fig. S1. (a) Pressure-composition phase diagram of the Mg-Si system. (b) and (c) Crystal structure of Mg_2Si.

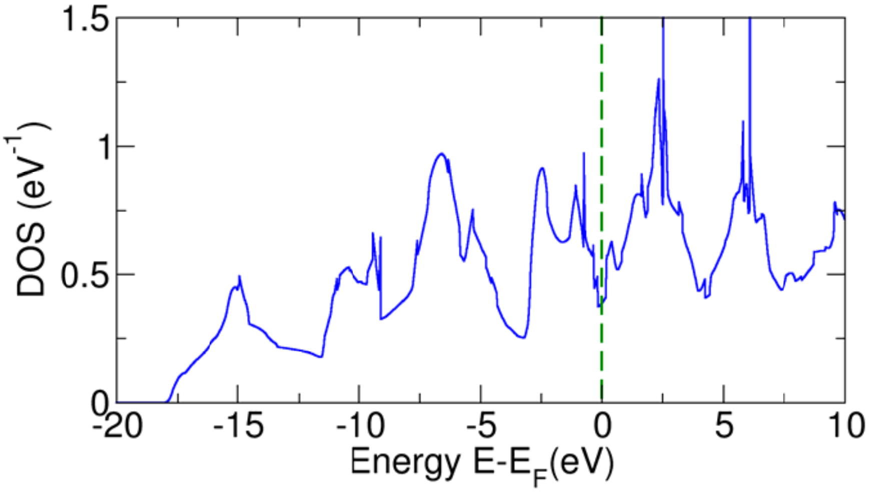

Fig. S2. The density of states (DOS) of Mg_2Si at 0.5 TPa.

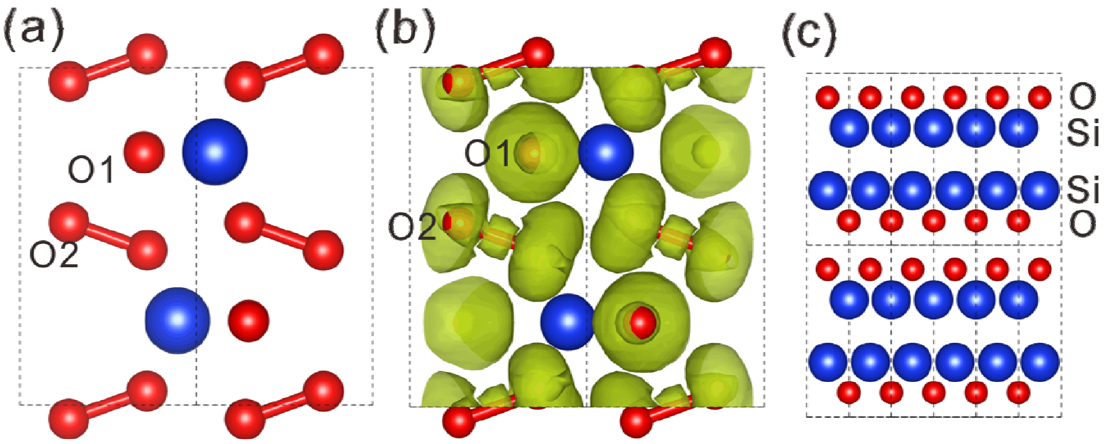

Fig. S3. (a) Crystal structure and (b) its corresponding isosurface of the electron localization function (ELF) of *mP*16-SiO_3 at 1.0 TPa with an isovalue of 0.65. O1 and O2 refer to two types of O atoms in *mP*16-SiO_3. (c) Crystal structure of *tP*4-SiO with Si-Si-O-O layered structure.

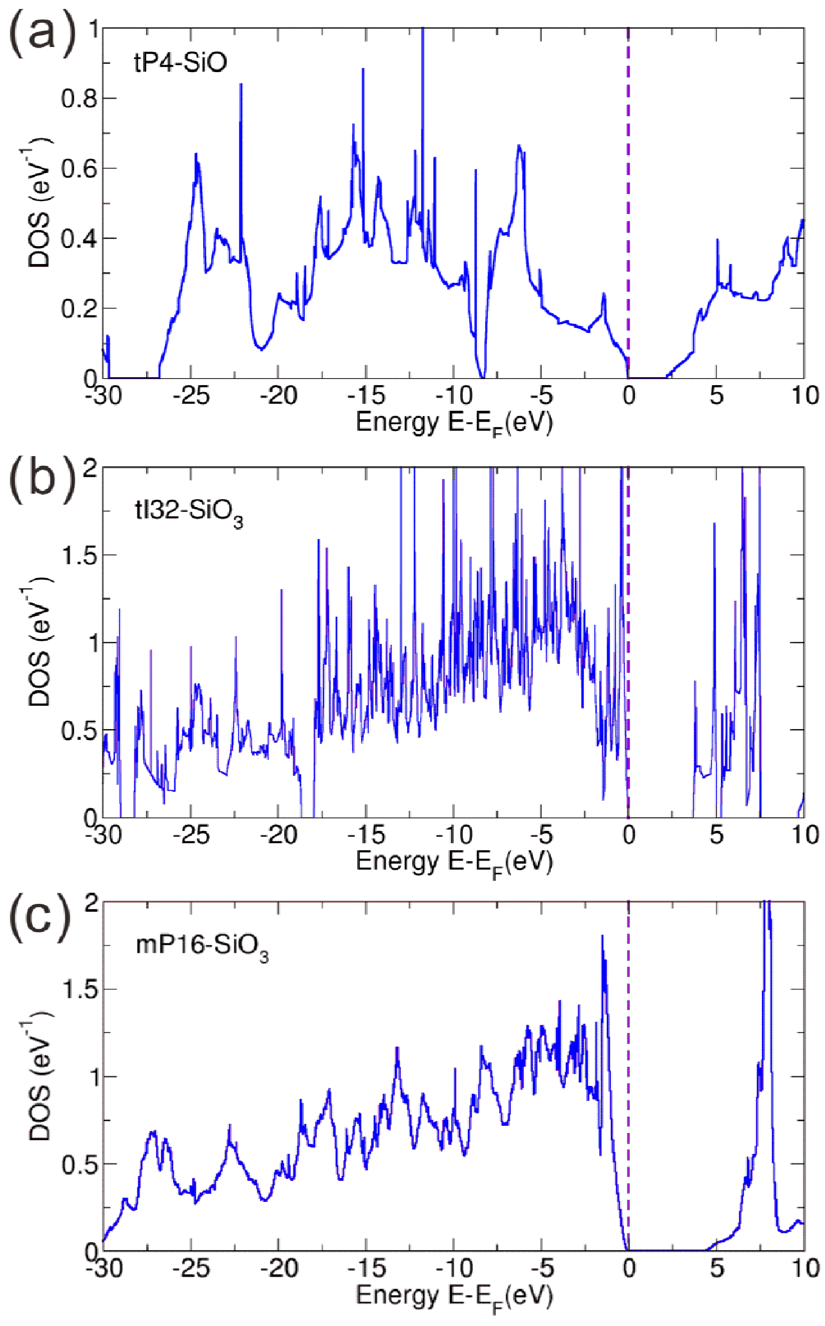

Fig. S4. The density of states (DOS) of *tP*4-SiO at 1.5 TPa, *tI*32-SiO_3 at 0.7 TPa, and *mP*16-SiO_3 at 1.0 TPa, respectively.

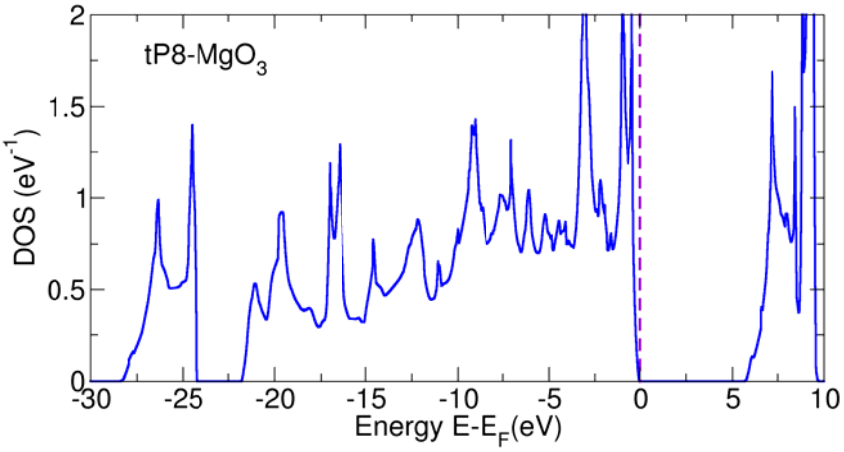

Fig. S5. The density of states (DOS) of *tP8*-MgO_3 at 1.0 TPa.

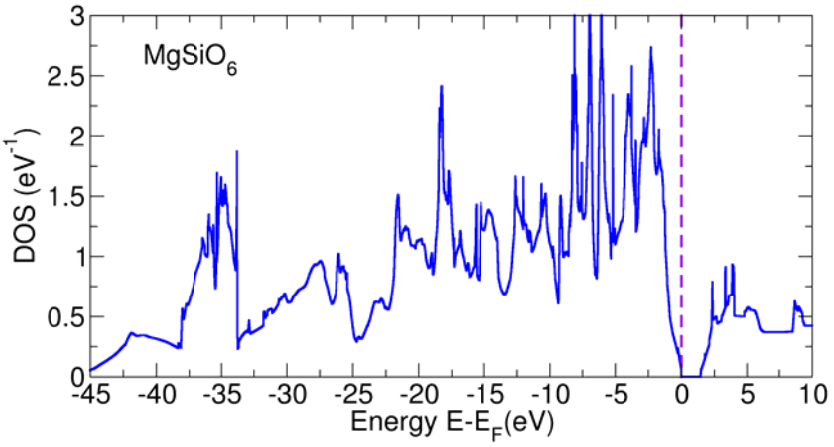

Fig. S6. The density of states (DOS) of *cp8*-$MgSiO_6$ at 3.0 TPa.

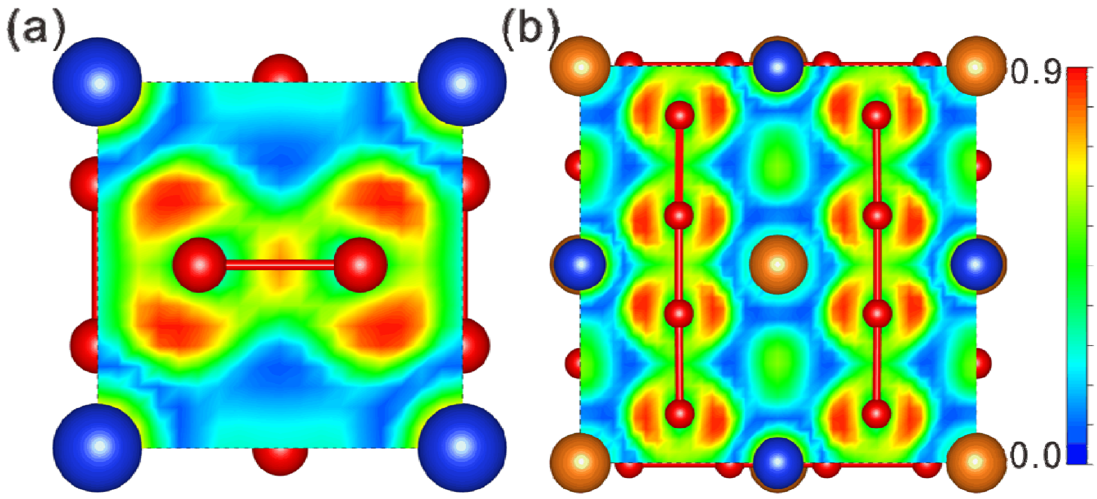

Fig. S7. Cross-section of the ELF of (a) *cP*8-$MgSiO_6$ and (b) *cF*64-$MgSi_3O_{12}$, respectively.
